# Determinants of survival in patients treated with CPX-351 for acute myeloid leukemia

**DOI:** 10.3389/fonc.2026.1813333

**Published:** 2026-04-27

**Authors:** Patrizia Chiusolo, Luana Fianchi, Filippo Frioni, Sabrina Giammarco, Elisabetta Metafuni, Maria Assunta Limongiello, Martina Quattrone, Marianna Criscuolo, Antonio Giordano, Simona Sica, Livio Pagano

**Affiliations:** 1 Section of Hematology, Department of Laboratory and Hematological Sciences, Catholic University, Rome, Italy; 2 Fondazione Policlinico Universitario A. Gemelli IRCCS, Rome, Italy

**Keywords:** acute myeloid leukemia, complex karyotype, CPX-351, HSCT, hematopoietic stem cell transplant, leukocytosis

## Abstract

CPX-351 for the treatment of acute myeloid leukemia (AML) has demonstrated better efficacy compared with standard chemotherapy regimens, with an excellent safety profile. We aimed to assess the determinants of response, relapse, and survival in patients treated with CPX-351 as the first-line therapy for AML. In this retrospective monocentric study, we analyzed 60 consecutive patients treated with CPX-351 for therapy-related AML or AML with myelodysplastic-related changes. We observed a 61% overall response rate, with 58% complete response. The presence of complex karyotype at diagnosis predicted lower response rates. Among patients who achieved complete response, the relapse rate was 50%, with a median relapse-free survival of 154 days. The median overall survival was 412 days. We found that the biological profile at diagnosis is associated with survival. Leukocytosis at diagnosis, complex karyotype, and mutations of *IDH1* and *SRSF2* were correlated with lower survival in the univariable analysis. In the multivariable analysis, leukocytosis and complex karyotype retained their statistical significance.

## Introduction

Acute myeloid leukemia (AML) is a heterogeneous neoplasia characterized by an increase of the clonal malignant myeloid precursor in the bone marrow and/or in peripheral blood (1). In the majority of cases, AML arises *de novo*; however, in a minority of patients, it can develop from previous myeloid neoplasms (2). It can be secondary to myeloproliferative diseases, typically myelofibrosis (in approximately 20% of cases) (3), or myelodysplastic syndrome (4). In some other patients, DNA damage from previous exposure to chemotherapy or radiation therapy for other neoplasms can cause AML (5–7).

CPX-351 is a liposomal combination of cytarabine and daunorubicin at a 5:1 molar ratio that is approved for the treatment of therapy-related acute myeloid leukemia (t-AML) and AML with myelodysplastic-related changes (AML-MRC) (8). The incidence of secondary AML increases with age, which has demonstrated worse outcomes in terms of response and survival than *de novo* AML (5, 9). CPX-351 has shown longer overall survival (OS) when compared with the standard 3 + 7 (cytarabine and daunorubicin) chemotherapy regimen in patients with high-risk AML aged 60–75 years (8), with a better safety profile (10). Lancet et al. demonstrated that CPX-351 treatment, compared with the standard 3 + 7 chemotherapy, had better median OS (9.56 *vs.* 5.95 months) and higher overall remission rate (47.7% *vs*. 33.3%), as well as in the analysis of age groups and AML subtypes (8). Moreover, allogeneic hematopoietic stem cell transplantation (HSCT) resulted feasible after CPX-351 treatment, as well as in older patients (11). While the efficacy and safety are better described, the determinants of response and the survival among high-risk patients treated with CPX-351 remain unknown. Some studies have addressed potential predictors of response looking at the molecular and cytogenetic characteristics, with non-univocal results (12–15). Chiche et al. found that the presence of *TP53* and *PTPN11* mutations was the only molecular variable that showed worse response rates. The authors also found monosomal karyotype to be a poor prognostic factor in the univariable analysis (12). The same results were not replicated by other authors (13–15). Guolo et al. showed that *TP53* mutation or other high-risk mutations according to the European LeukemiaNet (ELN) (16) were not prognostically detrimental (14). The impact of ELN genetic risk stratification in the setting of patients treated with CPX-351 largely remains to be understood: a recent multicentric Italian study showed that patients with *NPM1*-mutated AML benefited from treatment with CPX-351 and that allogeneic HSCT should be performed as soon as complete remission is achieved (17).

## Methods

### Study design

This retrospective monocentric analysis included 60 consecutive adult patients treated with CPX-351 as the first-line therapy for t-AML or AML-MRC from 2019 to 2024. All patients were treated according to the EMA/AIFA (European Medicines Agency/Italian Drug Authority) CPX-351 dosing schedule. We analyzed the impact of the clinical, biochemical, molecular, and cytogenetic characteristics of the patients on the response to induction therapy with CPX-351 and on the relapse-free survival (RFS) and OS. All clinical and biological information is available for the totality of patients and has been collected at the time of AML diagnosis. Allogeneic HSCT was up to the physician’s choice.

### Clinical, molecular, and cytogenetic variables

Clinical data included age, gender, performance status [classified according to the Eastern Cooperative Oncology Group (ECOG) performance status (18) and the Karnofsky performance scale (19)], the presence of myelodysplastic-related changes (defined as per the International Consensus Classification of Myeloid Neoplasms (20)), and, in the case of AML secondary to previous myelodysplastic syndrome, previous treatment with hypomethylating agents (HMAs). In the case of t-AML, we also took into account the type of previous chemotherapy regimen and previous radiation therapy, if administered. The biochemical parameters at diagnosis included anemia (defined as hemoglobin level <10 g/dl), severe anemia (defined as hemoglobin level <8 g/dl), thrombocytopenia (defined as platelet count <100 × 10^9^/L), severe thrombocytopenia (defined as platelet count <50 × 10^9^/L), leukocytosis [defined as white blood cell (WBC) count >10 × 1^9^/L], and hyperleukocytosis (defined as WBC count >50 × 1^9^/L). We defined leukocytosis as WBC >10 × 10^9^/L according to standard hematological definitions. The molecular characteristics of the patients were assessed with next-generation sequencing (NGS) analysis performed using the Illumina^®^ MiniSeq instrument on DNA extracted on bone marrow blood obtained at the time of diagnosis. The custom gene panel included the following genes: *RUNX1*, *TP53*, *EZH2*, *CUX1*, *REEP5*, *IKZF1*, *TET2*, *KIT*, *CBL*, *SF3B1*, *SRSF2*, *DNMT3A*, *ASXL1*, *IDH1*, *U2AF1*, *IDH2*, *CSF3R*, *PDL5*, *CBLB*, *NRAS*, *KRAS*, *ZRSR2*, *WT1*, *ETV6*, *JAK2*, *MPL*, *CALR*, *NPM1*, and *FLT3*. Core binding factor status assessment was performed using quantitative polymerase chain reaction (PCR). Bone marrow biopsy with immunohistochemical stain for *NPM1* was performed to determine its delocalization when mutated. After analyzing the molecular variables individually, we analyzed them in groups according to the ELN risk group (16) and Lindsley classification (21). Cytogenetic characteristics included both data from conventional cytogenetics and fluorescence *in situ* hybridization to detect myelodysplasia-related chromosome abnormalities (loss of chromosome 5q, 7q, or 17p material). We analyzed monosomal karyotype as an independent variable. For all patients, the cytogenetic and molecular variables were assessed at the time of diagnosis.

### Endpoint

The response criteria were assessed according to the International Working Group 2003 criteria (22) Complete remission (CR) included molecular CR and cytogenetic CR when a molecular or a cytogenetic marker, respectively, was present, but morphologic CR in the absence of a disease marker. The overall response rate (ORR) included CR, CR with incomplete hematologic recovery, and morphologic leukemia-free state (MLFS). All patients who achieved a response were assessed for minimal residual disease (MRD) using PCR, flow cytometry, and conventional cytogenetics according to the disease characteristics present at the time of diagnosis.

Relapse was defined as the presence of >5% of blasts in the cytomorphological or flow cytometry examination after having achieved a response. RFS was calculated from the time of induction treatment to the time of relapse for those patients who achieved a response. OS was calculated from the time of the induction to the time of death, or the last follow-up.

### Statistical analysis

RFS and OS were calculated with the Cox regression proportional hazards model. The ORR and the CR rate were calculated with the *χ^2^*. test. In the multivariable analysis, we took into account only the variables that, in the univariable analysis, had a *p* < 0.05. Confidence intervals (CIs) were computed with 95% coverage. Statistical analysis was performed using NCSS version 24.0.2.

### Ethical approval

The study was conducted in accordance with the Declaration of Helsinki and was approved by the Institutional Review Board of Policlinico Universitario Agostino Gemelli (protocol ID 4417; date of approval, October 6, 2021).

## Results

A total of 60 patients who received first-line chemotherapy with CPX-351 for previously untreated AML between 2019 and 2024 were included in the study. All patients were considered fit for intensive chemotherapy according to the SIE/SIES/GITMO criteria (23). The median age was 63 years (range, 46–76 years). Of the patients, 29 are women and 31 are men, and 23 patients were diagnosed with t-AML while 37 patients had AML-MRC. Among the 37 patients with AML-MRC, only seven patients received prior HMA. On the other hand, among the 23 patients with t-AML, 17 patients received chemotherapy for previous cancers and six patients received radiation therapy. According to the ELN genetic risk stratification, 41 patients were classified as high-risk AML, of which 14 had complex karyotype, and only 10 carried *TP53* mutation. In only six patients with the *TP53* mutation was a complex karyotype detected. Leukocytosis was present in 16 patients (WBC count range, 11–258 × 1^9^/L), while hyperleukocytosis was present in only seven patients (WBC count range, 51–258 × 1^9^/L). A total of 31 patients underwent allogeneic HSCT. Patients not subdued to allogeneic HSCT were considered frail according to the SIE/SIES/GITMO (23) criteria at the end of induction therapy and were consequently candidates to observational follow-up. The median follow-up for survivors was 474 days. **Table 1** summarizes the characteristics of the patients.

**Table 1:** Characteristics of the patients and results.

Total no. of patients	60
Median age (range)	63 years (46-76)
M/F	31/29
Diagnosis	t-AML: 23 patients (38%) AML-MRC: 37 patients (62%)
ELN risk stratification	High-risk: 41 patients (68%) Standard risk: 19 patients (32%)
Molecular profile	*TP53*: 10 patients (17%) *FLT3*: 2 patients (3%) *SF3B1*: 4 patients (7%) *ASXL1*: 6 patients (10%) *RUNX1*: 8 patients (14%) *U2AF1*: 4 patients (7%) *IDH*: 6 patients (10%) *EZH2*: 6 patients (10%) *SRSF2*: 3 patients (5%) *RAS*: 5 patients (8%) *ZRSR2*: 2 patients (3%) *DNMT3A*: 10 patients (17%) Other: 7 patients (12%)
Karyotipe	Normal: 35 patients (58%) 1 or 2 abnormalities: 11 patients (19%) Complex: 14 patients (23%)
ORR	37 patients (62%) CR: 35 patients (58%) MLFS: 2 patients (3%)
Relapse rate	50% (18 patients out of 37 achieving CR)
Median OS (range)	412 days (77–1904)
Univariable analysis on the determinants of OS	*IDH1* mutations (HR = 4, 95%CI = 1.3–12, *p* = 0.01) *SRSF2* (HR = 10, 95%CI = 2–50, *p* = 0.003) Leukocytosis at diagnosis (HR = 2, 95%CI = 1.5–10, *p* = 0.03) Complex karyotype (HR = 3.7, 95%CI = 1.6–9.2, *p* = 0.002)
Multivariable analysis on the determinants of OS	Leukocytosis (HR = 5.8, 95%CI = 3–21, *p* = 0.006) Complex karyotype (HR = 3.9, 95%CI = 2–15, *p* = 0.01)

*M*, male; *F*, female; *t-AML*, therapy-related acute myeloid leukemia; *AML-MRC*, AML with myelodysplastic-related changes; *ELN*, European LeukemiaNet; *ORR*, overall response rate; *OS*, overall survival; *CR*, complete remission; *MLFS*, morphologic leukemia-free state; *HR*, hazard ratio; *CI*, confidence interval.

The ORR was 61% (37 patients), including 35 CR and two MLFS. CR was achieved in 35 patients (58%): after one induction cycle in 25 (42%) patients and after the reinduction in 10 (17%) patients. Among the 35 patients who achieved MRD, only four patients showed MRD positivity. Only the presence of a complex karyotype at diagnosis was correlated with a lower ORR (OR = 10.2, 95%CI = 2.3–44, *p* = 0.001) and a lower CR rate (OR = 5, 95%CI = 1.2–16.2, *p* < 0.001) in the univariable analysis. We found that NGS- or PCR-detectable somatic mutations had no impact on ORR, with only the *TP53* mutations showing a trend (*p* = 0.06) despite not reaching statistical significance. Similarly, no mutation predicted failure to achieve CR. Even when clustered together according to the Lindsley classifier, we observed no impact of somatic mutations on the ORR and CR. In the same way, no clinical or biochemical variable registered at diagnosis was correlated with the ORR and CR in a statistically significant manner. The response rates were similar in patients with t-AML and AML-MRC (**Table 2**).

**Table 2:** Characteristics of the patients with AML-MRC (AML with myelodysplastic-related changes) and t-AML (therapy-related acute myeloid leukemia).

Characteristics of the patients	AML-MRC	t-AML
No. of patients	37 (62%)	23 (38%)
Median age (range)	66 years (46–76)	62 years (50–69)
M/F	16/19	9/14
ELN high-risk	26 (70%)	15 (65%)
Molecular profile	*TP53*: 4 patients (11%) *SF3B1*: 2 patients (5%) *ASXL1*: 5 patients (13%) *RUNX1*: 7 patients (17%) *U2AF1*: 4 patients (11%) *IDH*: 6 patients (15%) *EZH2*: 2 patients (5%) *SRSF2*: 3 patients (8%) *RAS*: 2 patients (5%) *DNMT3A*: 6 patients (15%)	*TP53*: 6 patients (26%) *SF3B1*: 2 patients (8%) *ASXL1*: 1 patient (4%) *RUNX1*: 1 patient (4%) *U2AF1*: 0 patients (0%) *IDH*: 0 patient (0%) *EZH2*: 4 patients (16%) *SRSF2*: 0 patient (0%) *RAS*: 3 patients (12%) *DNMT3A*: 4 patients (16%)
Complex karyotype	8 (21%)	6 (26%)
ORR	63%	60%
Relapse rate	50%	51%
Median OS (range)	399 days (77–1,904)	427 days (136–1,844)

*M*, male; *F*, female; *ELN*, European LeukemiaNet; *ORR*, overall response rate; *OS*, overall survival.

Relapse was observed in 18 patients (50% of patients achieving response) at a median time of 154 days (range, 64–1,049 days). We found no clinical, biochemical, molecular, or cytogenetic variable correlated with a lower RFS, with only the presence of a complex karyotype at diagnosis showing a trend (*p* = 0.08). No differences were observed in the relapse rates between the t-AML and AML-MRC subgroups (**Table 2**).

**Figure 1:**
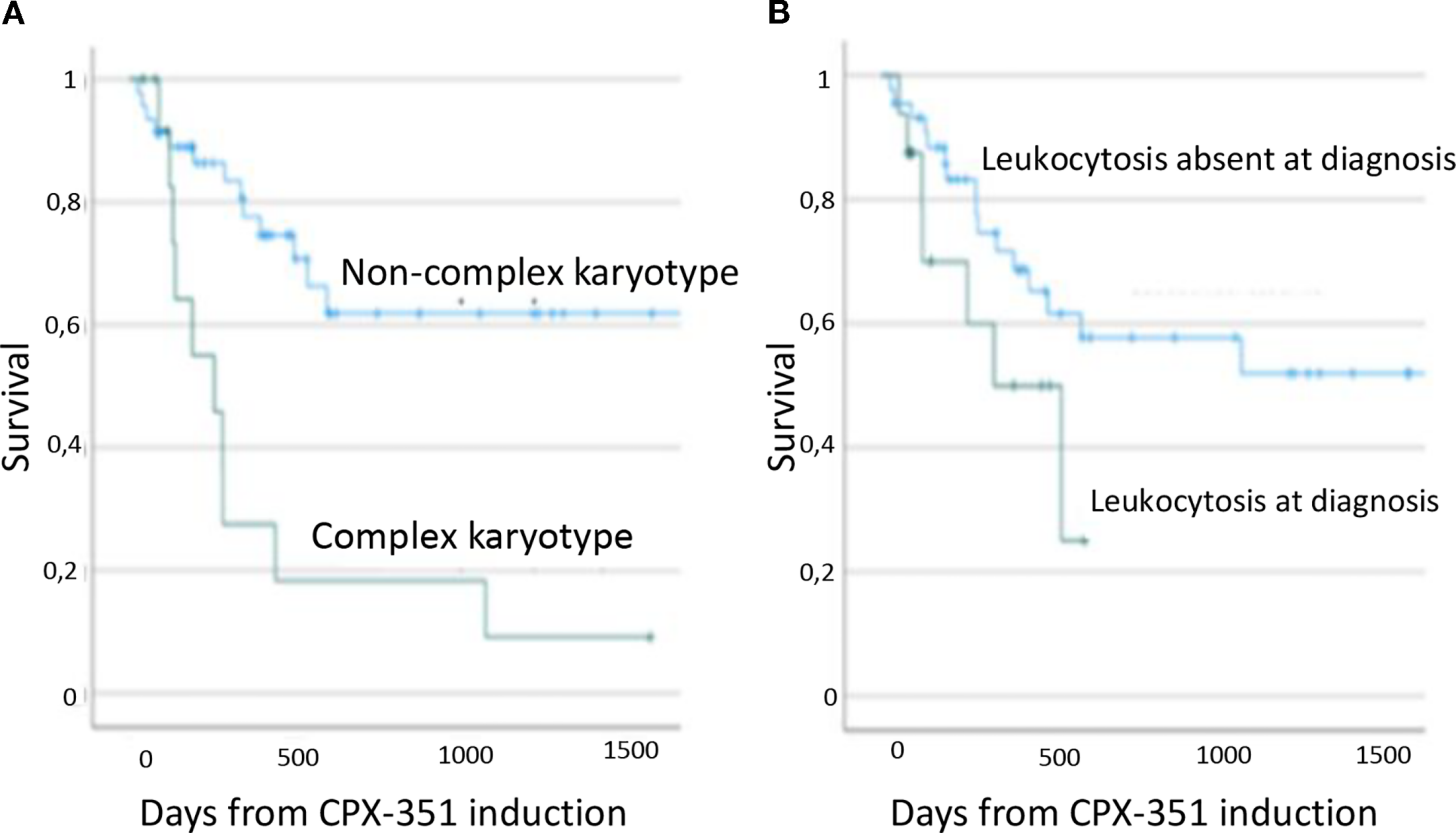
Impact of complex karyotype **(A)** and leukocytosis **(B)** on survival.

The median OS was 412 days (range, 77–1,904 days). The total number of deaths registered was 22. Univariable analysis of the factors correlated with OS showed that the clinical, cytogenetic, and molecular characteristics of patients impacted survival. Initial diagnosis (t-AML *vs*. AML-MRC) did not show an impact on OS (**Table 2**). Mutations of *IDH1* (HR = 4, 95%CI = 1.3–12, *p* = 0.01) and *SRSF2* (HR = 10, 95%CI = 2–50, *p* = 0.003), leukocytosis at diagnosis (HR = 2, 95%CI = 1.5–10, *p* = 0.03, median OS = 380 days *vs*. not reached), and the presence of a complex karyotype (HR = 3.7, 95%CI = 1.6–9.2, *p* = 0.002, median OS = 284 days *vs*. not reached) were correlated with a shortened OS. ELN and Lindsley classification failed to show an impact on OS. MRD positivity did not show an impact on survival given the small sample size. Patients undergoing allogeneic HSCT showed better survival compared with patients ineligible for HSCT (*p* < 0.001) when analyzed as a time-dependent variable with the Cox regression proportional hazards model (median OS = 146 *vs*. 565 days for patients ineligible for allogeneic HSCT and those undergoing HSCT, respectively). In the multivariable analysis, leukocytosis (HR = 5.8, 95%CI = 3–21, *p* = 0.006), a complex karyotype (HR = 3.9, 95%CI = 2–15, *p* = 0.01), and allogeneic HSCT (HR = 4.3, 95%CI = 2.5–6.3, *p* = 0.03) retained their statistical significance. **Figure 1** shows the Kaplan–Meier analysis on survival. The results are resumed in **Table 1**.

## Discussion

AML-MRC and t-AML represent high-risk subgroups of AML that, together, account for approximately 40% of newly diagnosed AML. These AML subgroups occur at an advanced age and show a dismal prognosis, worse than *de novo* AML (24, 25). In the open-label, randomized, phase III trial CLTR0310-301, CPX-351 showed better OS compared with treatment with the standard 3 + 7 chemotherapy (8). A number of studies have investigated the determinants of response and survival after CPX-351; however, the results remain conflicting (12–15).

In the first study addressing this topic, provided by Guolo et al. and including 71 patients, *TP53* mutation did not impact the OS or ORR after CPX-351 treatment (14). Similarly, Rautenberg et al. showed that somatic mutations of the *ASXL1*, *RUNX1*, and *TP53* genes were not correlated with lower ORR and OS (15). In a more recent multicentric study by Chiche et al., only *TP53* and *PTPN11* mutations showed a correlation with worse ORR in the multivariable analysis, while *TP53* mutation and a monosomal karyotype were correlated with a shortened OS in the univariable analysis, but not in the multivariable analysis. They also found splicing mutations as predictors of better survival (12).

In our monocentric study, we retrospectively analyzed 60 consecutive patients treated with CPX-351 as the first-line treatment for newly diagnosed t-AML and ALM-MRC. We collected all clinical, biochemical, molecular, and cytogenetic variables, all available at diagnosis, to determine their impact on the ORR, CR rate, RFS, and OS. We found that the molecular profile determined by NGS- and PCR-detectable somatic mutations was a poor determinant of response rate and survival compared with the presence of a complex karyotype. Only *IDH* and *SRSF2* showed an impact, but only in the univariable analysis and not in the multivariable analysis, given the low number of cases (six and three patients, respectively). Even when grouped together according to the ELN or Lindsley classification, the molecular profile resulted as not correlated with the ORR, RFS, and OS. Conversely, complex karyotype observed at diagnosis was correlated with a lower ORR, a lower CR rate, and a shortened OS in our study. It also showed a trend of determining a lower RFS, although did not reach statistical significance. Moreover, it was observed that leukocytosis at diagnosis was correlated with lower survival, and allogeneic HSCT should be performed in eligible patients.

Our findings are consistent with the literature, although it is extremely limited. When analyzing our cohort, we could not find a correlation between previous HMA therapy and better outcomes, probably due to the small number of patients having received prior treatment with HMA, i.e., only seven patients. Monosomal karyotype also failed to affect the outcomes, probably for the same reason, with only four patients diagnosed with monosomal karyotype AML without showing a complex karyotype.

## Conclusion

Although our study has some limitations, such as the retrospective design and the small number of patients included in our cohort, it highlights the prognostic impact of the complex karyotype at diagnosis in the setting of patients treated with CPX-351 as the major predictor of no response and shortened OS. Our study also shows how, in this particular setting of patients, the molecular characteristics of the patients may have a different prognostic weight compared with those treated with standard 3 + 7 chemotherapy. These data need to be validated in larger cohorts.

## Data Availability

The raw data supporting the conclusions of this article will be made available by the authors, without undue reservation.
